# Advances in organoid imaging and automated morphometric analysis: from optical microscopy to computational approaches

**DOI:** 10.3389/fcell.2026.1861934

**Published:** 2026-07-02

**Authors:** Anna V. Zubova, Ivan V. Simkin, Roman V. Shumilin, Dmitry A. Lifanov, Elizaveta S. Perepelitsa, Mikhail S. Sokolov, Nikita P. Kryuchkov, Stanislav O. Yurchenko, Alla B. Salmina, Sergey N. Illarioshkin

**Affiliations:** 1 Brain Science Institute, Russian Center of Neurology and Neurosciences, Moscow, Russia; 2 Bauman Moscow State Technical University, Moscow, Russia

**Keywords:** 3D-models, microscopy methods, morphometric post-processing of images, organoids, segmentation methods, spheroids

## Abstract

Researchers worldwide are increasingly interested in methods for reproducing organogenesis by culturing spheroids and organoids. Unlike spheroids, organoids are more closely related to organs in structure and functional activity. Due to the diversity of organoid production protocols and the lack of precise morphological criteria for standardizing these methods, there is a growing need to identify morphological features as significant distinguishing indicators of mature and functionally healthy organoids. Various semi- and automated programs have been developed to address these issues. This review examines programs for the morphometric analysis of spheroids and organoids. Most of these programs utilize images taken with a brightfield microscope and a confocal laser microscope.

## 1 Introduction

In recent years, there has been a growing number of publications on the production and application of three-dimensional multicellular *in vitro* models—spheroids and organoids. A distinctive feature of spheroids is that they consist of immortalized cell lines or primary culture cells, while organoids are formed from embryonic stem cells (ESCs), induced pluripotent stem cells (iPSCs), or somatic stem cells from healthy and diseased patients (Boot et al., 2024; Zhao et al., 2022; Lee et al., 2017). Organoids more accurately reproduce the physiological composition of the microenvironment of the corresponding tissues *in vivo* than spheroids, but the process of organoid formation is more complex and time-consuming. Therefore, spheroids are increasingly used for standard biophysical studies and high-throughput drug screening (Boot et al., 2024), while organoids, as revolutionary three-dimensional (3D) cell models, have become an indispensable tool in fundamental research in developmental biology, as well as in disease modeling, due to their ability to self-organize and mimic the structure and functions of real organs (Lukonin et al., 2020). Thus, organoids reproduce organ-like structures—miniature versions of organs grown *in vitro*. To date, such mini-organs as intestinal organoids (Cieza et al., 2021), kidney organoids (Huang et al., 2020), tumor organoids (Huo et al., 2023), breast organoids (Cairns et al., 2020), lung organoids (Pauli et al., 2017), and cerebral organoids (Lancaster et al., 2013) have already been obtained. Cerebral organoids derived from iPSCs have particular research and practical value, as they provide the ability to reproduce specific features of human brain tissue that cannot be studied *in vivo* in animal models. Due to their physiological proximity, cerebral organoids derived from iPSCs represent more relevant models of various neurological and neurodegenerative diseases. For example, post-mortem histological brain samples represent the terminal stage of the disease and therefore do not allow us to trace the mechanisms of neurological disease development at the cellular level. Furthermore, ethical restrictions hinder brain tissue biopsy (Nam et al., 2020). Eichmüller and Knoblich note that animal models, used in many studies, also cannot provide a comprehensive source of information on disease pathogenesis, as the mechanisms of development in humans and animals are not always identical (Eichmüller and Knoblich, 2022). In contrast, iPSC-derived organoids are complex, patient-specific models consisting, like tissues, of different cell types or cells at different stages of maturity, allowing to reproduce *in vitro* the pathogenetic processes characteristic of humans.

The field of organoids research is advancing at an unprecedented pace. The transition from subjective visual assessment to objective quantitative analysis is fundamental to improving the reproducibility, scalability, and statistical significance of research. The constant emergence of new imaging technologies and software tools necessitates the systematization and critical evaluation of existing knowledge. The existing diversity of microscopy methods and software solutions for morphometric analysis, each with its own unique advantages and disadvantages, requires in-depth analysis. Such analysis will enable researchers to make informed decisions when selecting the most appropriate tools for specific tasks. This review is also necessary to identify current gaps, unresolved issues, and limitations in the field of organoid morphometric analysis, which can serve as a starting point for future innovative research and technological developments.

The review included publications describing visualization methods directly or potentially applicable to the analysis of three-dimensional (3D) and four-dimensional (4D) cellular models. Particular attention was given to articles containing specific quantitative data on the performance of the methods (e.g., segmentation accuracy, processing speed) and a detailed description of the software functionality. Both specialized tools developed specifically for organoid analysis and more general bioimage analysis platforms that can be successfully adapted for organoid morphometry were considered.

Obtaining high-quality images is only the first step in the morphometric analysis of organoids. Extracting quantitative data requires automated or semi-automated software that can segment objects (highlight their boundaries), measure various morphological parameters, and analyze their dynamics. The development of 3D and 4D cell models has led to an exponential increase in data complexity and the demands on visualization and analysis methods. This, in turn, has created a fundamental challenge for morphometric methods, as simple 2D measurements have become insufficient to characterize complex three-dimensional structures and their changes over time. Thus, there is a need to develop new, more powerful microscopic approaches and computational algorithms capable of processing volumetric and temporal data.

The purpose of this literature review is to examine protocols for obtaining spheroids and organoids, to investigate methods for visualizing 3D models, and to analyze modern software tools for morphometric analysis, identifying their key characteristics, advantages, disadvantages, and areas of application. We examined these aspects for various types of spheroids and organoids, focusing on our research subjects of immediate interest—cerebral organoids. To achieve this goal, the following objectives were set:

1. To describe methods for obtaining and visualizing spheroids and organoids, as well as methods for analyzing the resulting images (specialized software solutions and platforms for segmentation and quantitative morphometric analysis of organoid images).
2. To conduct a comprehensive comparative analysis of the reviewed methods and tools, identifying their strengths and weaknesses, as well as specific application areas.

## 2 Characteristics of organoids and assembloids production methods

The earliest publication (2001) on the differentiation of neurons from human pluripotent stem cells was an article on the formation of neural rosettes from embryoid bodies, and the first publication (2013) on the production of cerebral organoids from human pluripotent stem cells was the work of a group of scientists led by Lancaster et al. (2013). Lancaster et al. stimulated the differentiation of embryoid bodies in the neuroectodermal direction in Matrigel using a rotating bioreactor. Moreover, Y.J. Hong et al. developed a protocol for the production of cerebral organoids from human induced pluripotent cells and from human embryonic stem cells (Hong et al., 2022). Thus, a variety of protocols have been developed for the creation of 3D structures from iPSCs obtained from patient skin fibroblasts or peripheral blood mononuclear cells. Organoid production protocols vary in terms of medium composition and technological aspects, and the organoids themselves differ in the representation of different cell types (Boot et al., 2024). Over the past decades, methods have been developed for producing cerebral organoids capable of reproducing specific brain regions: the hippocampus (Pomeshchik et al., 2020), cerebellum (Ballabio et al., 2020), midbrain (Jo et al., 2016), pituitary gland (Kano et al., 2023), thalamus (Fligor et al., 2021), and cerebral cortex (Eiraku et al., 2008; Lancaster et al., 2013; Paşca et al., 2015).

One of the most common production methods, suspension culture, is being modified and improved. Standardization of embryonic body differentiation was achieved using low-adhesion U-shaped plates, microarrays, and the hanging drop method. A bioreactor and orbital shaker were used during the agitation stage of cerebral organoids (Shnaider, 2018). In 2017, Lancaster et al. used bioengineered PLGA (poly(lactic-co-glycolic acid)) microfibrils to create a floating scaffold mimicking the cortex’s basement membrane. This approach promoted improved neuroectodermal differentiation and proper formation of cortical tissue cytoarchitecture compared to the standard protocol (Lancaster et al., 2017).

Laboratory-produced three-dimensional brain organoids have become alternatives to classical animal and *in vitro* models. Cerebral organoids have become a convenient tool for patient-specific modeling of hereditary and sporadic forms of neurological, neurodegenerative and neuropsychiatric diseases, including Alzheimer’s disease (Yan et al., 2018), Parkinson’s disease (Kim et al., 2019), microcephaly caused by Zika virus infection (Krenn et al., 2021), schizophrenia (Ye et al., 2017), autism spectrum disorders (Mariani et al., 2015).

The cellular composition of brain organoids matches that of human brain tissue, consisting of neurons, glia, and microglia. The structure of organoids is similar to some regions of the human brain, making them ideal for studying intercellular interactions between neurons and glia (Hong et al., 2022).

Organoids contain intermediate progenitor cells and neuronal cells at various stages of differentiation (Salmina et al., 2021). Astrocytes (Yakoub, 2019) and microglia (Ormel et al., 2018) can spontaneously arise in organoids. Using proteomic and functional analysis, Dezonne et al. demonstrated that astrocytes isolated from organoids have similar functional pathways to those in the human cerebral cortex (Dezonne et al., 2017). Dendritic spines and neural networks were also observed to appear in the organoids when cultured for more than 9 months (Quadrato et al., 2017).

**FIGURE 1:**
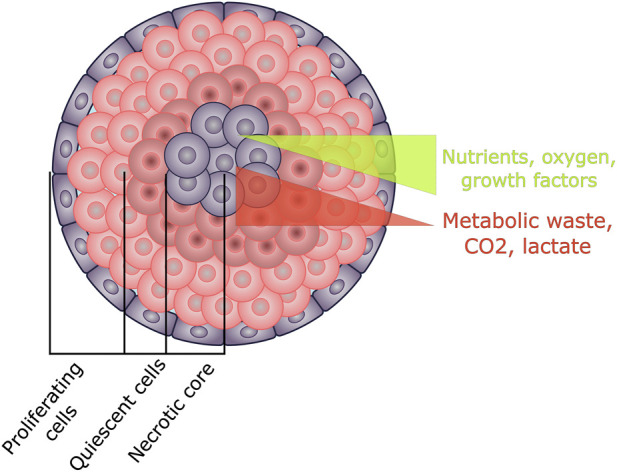
Average structure of spheroids and organoids. The main spatial zones observed during 3D cultivation are shown. Proliferating cells are the most metabolically active part of the cells; they have the greatest access to dissolved oxygen, nutrients, and signaling factors. This part of the cells enables the growth (and, under certain conditions, migration) of organoids and spheroids. Quiescent cells are resting cells; their level of metabolism, proliferation, and oxygen consumption is reduced due to reduced access to oxygen and nutrients. Upon exfoliation of the upper cell layers or migration to the matrix, these cells are able to re-increase their metabolic rate. Necrotic core is the inner part of spheroids and organoids (provided that they are not hollow). This part of the 3D structure contains virtually no living, metabolically active cells; most of the contents of this zone are cellular debris following necrosis or apoptosis.

The structure of cerebral organoids is still imperfect for several reasons: due to the lack of a developed vascular system, the central part of the organoid becomes necrotic (Figure 1), and even after long-term culture for 2 months, cerebral organoids are comparable only to the intrauterine level of brain development (Logan et al., 2020). It has been established that cells in a 3D structure have a number of differences from cells forming a monolayer: shape, proliferation rate, pharmacological resistance, degree of differentiation, and gene expression (Sogomonyan et al., 2022).

Many research groups are already developing and exploring approaches to promote the maturation of organoid cells and interactions between them. One such approach is the generation of assembloids. Assembloids are formed by integrating multiple organoids (multiregional structures) or by fusing organoids with additional cell types (Kanton and Paşca, 2022). For example, Fligor et al. created an assembloid model of the visual system incorporating cortical and thalamic organoids. Visual projection was modeled through the response of retinal glial cells to environmental cues. Furthermore, the survival of retinal cells in these assembloids was increased compared to retinal organoids (Fligor et al., 2021).

Upon fusion of medial ganglionic eminence and cortex-specific organoids, migration of ventral interneurons from the ganglionic eminence organoid into the cortex organoid was observed. This study provided the opportunity to analyze interneuron migration and integration in real time for further modeling and study of interneurogenesis in brain organoids (Xiang et al., 2017). Another approach to functional maturation of organoids is their transplantation into the animal brain. Mansour et al. implanted cerebral organoids cultured for 40–50 days into the retrosplenial cortex of immunodeficient mice. Organoid engraftment was confirmed to occur within 2–12 weeks, with neuronal cell maturation, gliogenesis, microglial integration, and organoid vascularization observed (Mansour et al., 2018).

Ham et al. successfully addressed the issue of cerebral organoid vascularization by inducing the differentiation of vascular endothelial cells within them under the influence of VEGF. This was confirmed by the positive expression of the endothelial cell marker CD31 and the tight junction marker characteristic of the BBB, claudin-5 (Ham et al., 2020). The next equally successful method for forming a vascular system in organoids was the co-cultivation of ESCs or iPSCs with human umbilical vein endothelial cells (HUVEC) (Shi et al., 2020). It was noted that a high concentration of VEGF stimulates the differentiation of endothelial and neuronal cells in cerebral organoids. The formation of vascular tubes with a bilayer structure is facilitated by culturing cerebral organoids with VEGF and Wnt7a (Ham et al., 2020). Another approach to organoid vascularization is the fusion of separately grown neural and vascular organoids (Wörsdörfer et al., 2019). As a result, fusion in cerebral organoids resulted in the formation of a vascular structure with an increased number of neural progenitors, which may support the hypothesis that neurogenesis is regulated by vessels. These hybrid organoids contained microglia, and direct contacts between endothelial cells and glial cells, which are important *in vivo* for the formation of the blood-brain barrier, were observed. The researchers note that such organoids may serve as a model for neurovascular interactions (Sun et al., 2022). Ahn et al. also reported the formation of a vascular network within a cortical organoid as a result of its fusion with vascular cells (Ahn et al., 2021). In addition to these methods, microfluidic technologies exist for the vascularization of cerebral organoids, for example, using 3D-printed microfluidic chips (Salmon et al., 2022).

Thus, organoids already allow researchers to study complex biological processes to varying degrees in controlled conditions, overcoming the limitations of traditional two-dimensional (2D) cell cultures, which are unable to fully reproduce the complexity of cellular interactions and tissue architecture *in vivo* (Lukonin et al., 2020).

## 3 The importance of organoid imaging

**FIGURE 2:**
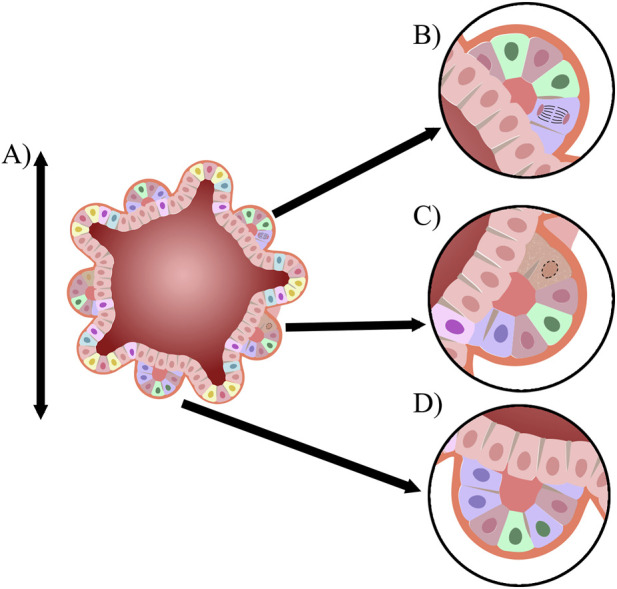
Key morphological features of organoids and spheroids considered in morphometric analysis: **(A)** Shape and size **(B)** Proliferating cells **(C)** Dead/dying cells **(D)** Cell types.

Organoids, as promising tissue-engineering tools, require suitable imaging methods (Morris and Payne, 2019) and accurate quantitative morphometric analysis. Such methods allow for the objective assessment of key parameters such as growth, differentiation, responses to various external stimuli (e.g., drugs), and the development of pathological changes (Figure 2).

Many programs for morphometric analysis are also capable of detecting not only the presence of the features listed in points B, C, and D, but also quantifying them and accounting for their spatial distribution.

## 4 The importance of morphological analysis of organoids

**FIGURE 3:**
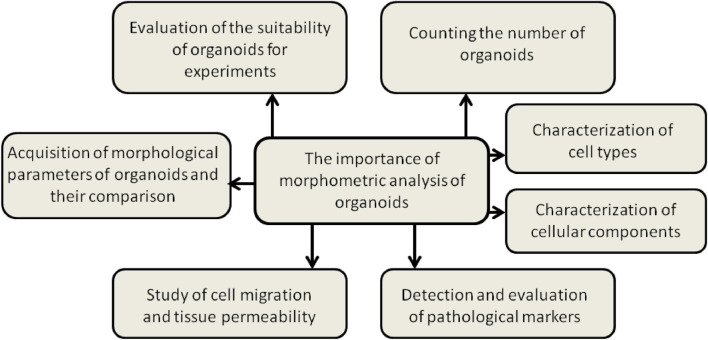
Applications of organoid morphometric analysis: cell counting, characterization of cell types (stem and proliferating cells, neuronal populations, oligodendrocytes, astrocytes, microglia, epithelium) and cellular components (nuclei, mitochondria, synapses, dendrites, or axons); search for pathological markers, study of cell migration, investigation of tissue permeability and manifestations of necrosis; obtaining morphometric parameters of organoids and comparison of organoid groups with each other.

One of the key indicators of a mature, functionally healthy organoid is its morphology. This important parameter allows for the assessment of organoid quality (Kahveci et al., 2025). For example, changes in organoid morphology at early stages and the formation of budding structures serve as key indicators of successful brain organoid differentiation. To optimize morphological analysis, image-capture microscopy methods and various image analysis software are used. Morphometric analysis is necessary not only for cell counting and type identification but also for research purposes. The importance of morphometric analysis of organoids is clearly illustrated in Figure 3.

## 5 Spheroids and organoids imaging methods

### 5.1 Culture viability assessment

To analyze cells in spheroids, some of the techniques used for analyzing conventional cell cultures can be used (Danilova et al., 2021). In general, viability assessment methods can be divided into those that analyze the medium for the presence of cell death markers or dissolved colored compounds, and those that analyze the properties and staining of the entire spheroid. For example, the former include the LDH test (Cox et al., 2021), the resazurin test, assessment of the leakage of fluorescent proteins from dead cells (Lifanov et al., 2025), and assessment of the leakage of fluorescent dyes. The latter group includes a modification of the MTT or WST8 test followed by an assessment of the staining intensity of the entire spheroid *in situ* (Popova et al., 2022) without solubilizing the colored or fluorescent reaction products; evaluation of fluorescence in the green and red channels using dyes that penetrate and do not penetrate living cells (e.g., calcein AM/propidium iodide) (Saydé et al., 2024); measurement of total bioluminescence using appropriate reporter systems. W. Senkowski et al. utilized a rather mechanistic yet robust approach, using GFP under the CMV promoter as a non-specific indicator of the metabolic status of cells (specifically, protein synthesis processes), and assessed the cytotoxic effect of the tested promising anticancer agents based on the level of total spheroid fluorescence. Interestingly, in the same work, it was once again shown that some promising cytostatic drugs, which showed good activity against cancer cells in a 2D monolayer, did not exhibit their effect when moving to a 3D culture (Senkowski et al., 2015).

Camarero et al. demonstrated the effect of laser radiation with wavelengths of 808 and 1,450 nm (near-infrared) on spheroids formed by epithelial cells of adenocarcinoma (an example of a tightly formed spheroid) and glioblastoma (an example of a spheroid with a large intercellular volume). Mature spheroids formed after laser irradiation changed in size, which correlated with spheroid viability. This allowed them to determine the irradiation thresholds for spheroid survival in laser microscopy experiments (Camarero et al., 2023). Thus, changes in the size of 3D objects can serve as an indirect indicator of the viability of their constituent cells.

### 5.2 Pre-treatment of organoids for imaging

Three-dimensional brain cultures, including cerebral organoids, are studied both whole-mount and at the molecular and cellular levels. Most often, microscopic examination is used to study cerebral organoids, followed by subsequent analysis using a variety of methods, most commonly computer processing. Cerebral organoids are first studied whole, in cleared form, and then in sliced form (Brémond et al., 2021).

#### 5.2.1 Organoids preparation for study as a whole sample

Optical clearing methods are used to study three-dimensional whole specimens, as it is important to reduce light scattering to ensure the refractive indices of various cellular components are consistent when imaging large three-dimensional structures (Dekkers et al., 2019). Optical clearing allows the specimen to be made transparent using various solvents (organic solvents, highly refractive aqueous solutions, tissue hyperhydrating and transforming solutions). These protocols were developed for studying brain structures, but have also been applied to other organs and are used to study organoids (Matryba et al., 2019). Protocols also exist for clearing organoids while preserving their physiological functions and viability—for example, the isotonic clearing medium SeeDB-Live, described by Inagaki S. et al. (Inagaki et al., 2024). A mixture of fructose and glycerol was used to clear live organoids from the respiratory tract, colon, kidney, liver, and mammary gland of humans and mice. The authors of the article note that this clearing protocol is applicable to other types of organoids as well (Dekkers et al., 2019).

#### 5.2.2 Organoids sections preparation

Microscopic examination of three-dimensional brain structures requires preliminary preparation: fixation in paraformaldehyde to stop enzymatic reactions and cutting three-dimensional structures into sections of 5–50 μm thickness to facilitate visualization of cerebral organoids and spheroids (Mansour et al., 2018).

### 5.3 Microscopic methods for studying cerebral organoids

**FIGURE 4:**
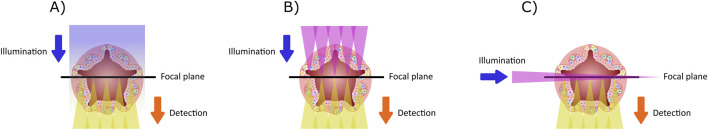
The most common types of microscopy and a schematic representation of their principles. **(A)** Light microscopy–the most common type of microscopy. It can use either white light or light with a narrow spectral range (for fluorescence microscopy). **(B)** Confocal microscopy–most often used in fluorescence microscopy. Unlike light microscopy, images outside the focal plane are not captured by the detector (they do not pass through the pinhole). Often, when examining 3D objects, it is necessary to either physically section the spheroid or organoid being examined, or clear it with special solutions (see Section 5.2.1. Organoids preparation for study as a whole sample). **(C)** Light sheet microscopy–a type of microscopy in which illumination occurs from the side and the light beam is shaped like a “sheet”. It is also typically used in conjunction with fluorescent staining. Unlike confocal microscopy, there is no need for a pinhole, and light sheet microscopy makes it easier to reconstruct an object in 3D.

Most researchers use brightfield, light sheet, and confocal microscopy to address the diverse needs of cerebral organoid analysis (observation, morphometric analysis, and quantification) (Brémond et al., 2021). Less than 2% of studies utilize inverted and phase-contrast microscopes (Thorn, 2016). The principles of image formation in different types of microscopy are shown in Figure 4.

#### 5.3.1 Confocal laser microscopy

A confocal microscope is used more often than other fluorescence microscopes for visualizing organoids, as confocal microscopy allows for the study of organoids in 3D space, unlike light microscopy. However, unlike brightfield microscopy, which can preserve the original color of the specimens being studied, confocal microscopy requires the use of fluorescent labels (Monzel et al., 2017). Confocal microscopy can also be used to obtain images without slicing organoids: for example, a method has been developed that allows for growing organoids up to 100 μm thick (Durens et al., 2020).

Three-dimensional visualization offers the most effective means for obtaining shape information and quantifying three-dimensional cellular structures compared to other methods. Härmä et al. obtained confocal images of three-dimensional organotypic cultures of 12 prostate and breast cancer cell lines using a confocal microscope. These images were then processed using the AMIDA program described below. Using these combined methods, three drugs were identified that inhibit metastasis development without inhibiting proliferation and apoptosis in non-metastatic tissue (Härmä et al., 2014).

#### 5.3.2 Bright field microscopy

In a study by Tomas et al., brightfield microscopy using the Incucyte® S3 organoid module, immunohistochemical staining, and immunofluorescence were used to compare the morphology of epithelial ovarian cancer spheroids and organoids (Tomas et al., 2025). These methods revealed that organoids, unlike spheroids, were morphologically heterogeneous and differed in growth dynamics and proliferative activity (Tomas et al., 2025).

Organoids grown using hydrogels cannot be aligned in the same focal plane, resulting in images in transmitted light with uneven blurring even at low magnification and low aperture values. Furthermore, the hydrogel medium contributes to distortion of phase and differential contrast images due to spherical aberration and brightfield noise (Borten et al., 2018). These problematic aspects were addressed using the OrganoSeg automated system, which is capable of processing such images. This method is described below.

Thus, confocal laser microscopy is optimal for obtaining images of 3D objects, but requires the use of fluorescent labels. At the same time, brightfield microscopy is a more versatile tool for working with organoids, allowing not only to obtain organoid images for subsequent analysis but also to conduct observations at different stages of organoid development without special preliminary sample preparation. Below, we review programs for processing images obtained primarily using brightfield microscopy (OrganoSeg, AnaSP, BraIn, OrganoLabeler, OrganoID, Label Free Tracker, Trackmate, Elephant) and confocal laser microscopy (Fiji with ImgLib, Organoid Tracker, Trackmate). Some of the programs can process both types of images (Ilastik, AMIDA, MOrgAna). Some of these programs can also process organoid images obtained using phase-contrast microscopy (OrganoSeg, OrganoID). Light sheet fluorescence microscopy (LSFM) is also used to image 3D structures, but it is only used in 3% of organoid observation studies due to the high cost of an LSF microscope and sample preparation requirements (Brémond et al., 2021). Therefore, it is not considered in this review. Table 1 presents the microscopy methods and associated image processing software.

**TABLE 1:** Microscopy methods and automated programs for obtaining and processing images of organoids and spheroids.

The automated program	Microscopy methods	Analyzed Features	Examples of organoids and spheroids	Automatization	User complexity	Score*
OrganoSeg	Bright field microscopy	Area, Convex area, Major axis length, Diameter, Perimeter, Minor axis length, Solidity, Extent, Correlation, Std. Dev. Pixel intensity, Pixel coefficient of variation, Contrast, Skewness, Eccentricity, Perimeter-to-area ratio, Zernike moment: amplitude, Mean pixel intensity, Energy, Homogenity, Orientation, Kurtosis, Zernike moment: imaginary, Zernike moment: phase, (total number 23)	colorectal cancer organoid (Farin et al., 2023)	Automated (Semi-Automated for overlapping organoids)	Average	2 + 1+2 + 2 (7)
			pancreatic cancer organoids (Grossman et al., 2022)			
			intestinal organoids (Montes-Olivas et al., 2023)			
			brain organoids (Schröter et al., 2024)			
			lung cancer spheroids (Piccinini, 2015)			
			pancreatic, lung, colon, and adenoid cystic carcinoma organoids ( Matthews et al., 2022)			
MOrgAna	Bright field microscopy	Area, Eccentricity, Major axis length, Perimeter, Minor axis length, Equivalent diameter, Euler number, Extent, Orientation, Locoefa coefficient (total number 10)	human somitoids (organoids of epithelial somite-like structures) (Sanaki-Matsumiya et al., 2022; Yaman and Ramanathan, 2023)	Semi-automated	Low	1 + 2+1 + 2 (6)
OrganoLabeler	Bright field microscopy	Only segmentation	brain organoids (Kahveci et al., 2024)	Automated	Low	2 + 2+0 + 2 (6)
Label Free Tracker	Bright field microscopy Confocal microscopy in bright field	(4D) Cell number, Cell Volume (total number 2)	breast adenocarcinoma organoids (Tebon et al., 2023), intestinal organoids (Kok et al., 2025)	Semi-automated	Average	1 + 1+1 + 2 (5)
OrganoID	Bright field microscopy; phase contrast microscopy	Area, Perimeter, Circularity, Solidity, Eccentricity (total number 5)	pancreatic, lung, colon, and adenoid cystic carcinoma organoids (Matthews et al., 2022)	Automated	Average	2 + 1+1 + 2 (6)
AnaSP	Bright field microscopy Immunofluorescence and light sheet fluorescence microscopy (LSFM)	Major axis length, Minor axis length, Equivalent diameter, Perimeter, Area, Volume, Sphericity (total number 7)	liver, stomach, and intestinal cancer organoids (Shi et al., 2024), 3D tumor spheroid, tumor spheroids of MRC-5 cell line (Zanoni et al., 2016)	Automated	Low	2 + 2+1 + 2 (7)
Elephant	Light sheet microscopy, fluorescence microscopy	Only segmentation	human intestinal organoids (Sugawara et al., 2022)	Semi-automated	High	1 + 0+0 + 1 (2)
Ilastik	Phase contrast microscopy, Confocal microscopy	Only segmentation	human axial organoids (Yaman and Ramanathan, 2023)	Semi-automated	Low	1 + 2+0 + 1 (4)
AMIDA	Confocal microscopy	Area, Circularity, Roughness, Density, FiltRound, RoundDiff, AppIndex, MaxApp, MedApp, AppNumber, Deviation, Closest, Neighbors, SharedBound, AreaRatio, Hollowness, CellNumber, AveArea, CellRatio, (Center of mass, Radius are not included in the result file) (total number 21)	prostate and breast cancer spheroids (Härmä et al., 2014; Murillo-Garzón et al., 2018)	Automated	Average	2 + 1+2 + 1 (6)
			triple-negative breast cancer organoids (Petruk et al., 2021)			
Fiji	Confocal microscopy	Depend on the plugins used	human somitoids (Yaman and Ramanathan, 2023)	Semi-automated	Low	1 + 2+2 + 1 (6)
SpheroidAnalyser	Confocal microscopy	Area, Diameter, Circularity, Volume, Perimeter (total number 5)	2D, 3D structures of tumor cells (Barrow et al., 2022)	Automated	Low	2 + 2+1 + 1 (6)
BraIn	Confocal microscopy	Area, Feret diameter, Perimeter, Circularity, Roundness (total number 5)	brain organoids (Kahveci et al., 2025)	Automated	Low	2 + 2+1 + 1 (6)
Organoid Tracker	Confocal microscopy	Only cell tracking in 4D	intestinal organoids (Kok et al., 2020)	Semi-automated	Average	1 + 2+0 + 1 (4)
CellProfiler	Mostly fluorescence microscopy	Depend on the module used	hiPSC, mouse embryo blastocyst, trophoblast stem cells, etc. (McQuin et al., 2018)	Automated	Low	2 + 2+2 + 1 (7)

* Score = A (Automatization) + C (User complexity) + F (Number of Analyzed Features) + M (The accessibility of the microscopy method). For more information follow Table 2.

## 6 Evaluation of methods for the segmentation and morphometric analysis of spheroids, organoids images and additional tools for cells analysis

### 6.1 OrganoSeg

OrganoSeg is a software for automated organoid morphometry using brightfield microscopy. Its easy-to-use interface reduces the learning curve for users without specialized IT training. According to the authors' Kolmogorov-Smirnov test, the program demonstrates high accuracy and scalability, outperforming CellProfiler and ImageJ in organoid segmentation. OrganoSeg has successfully processed over 10,000 organoids in studies of breast cancer (5,167 spheroids) and colorectal cancer (5,743 organoids). The program provides multiparametric analysis, extracting 23 morphometric and textural parameters, such as contrast, area, diameter, orientation, eccentricity, and others, and uses tSNE for phenotype classification, revealing biological processes. OrganoSeg is open source and compatible with brightfield, phase contrast, and DIC imaging. OrganoSeg’s drawbacks include its technical complexity, requiring parameter optimization (e.g., local adaptive thresholding), and low throughput for out-of-focus organoids in 3D matrices. Its suitability for non-standard organoid shapes is limited, potentially leading to erroneous results. The accuracy of the results is highly dependent on the quality of the source images. There is a risk of false positives and false negatives, as well as high computing power requirements for processing large volumes of data. Despite automation, the final results often require human review to confirm the accuracy of the analysis. OrganoSeg demonstrates how automation and scalability are becoming key factors in the morphometric analysis of organoids, enabling researchers to move from labor-intensive manual analysis to high-throughput screening. The ability to process thousands of samples and extract multiple parameters significantly accelerates research, particularly in the fields of drug screening and disease modeling. However, as with any automated system, its effectiveness is highly dependent on the quality of input data and requires some skill to set up, highlighting the continued need for human supervision and verification (Borten et al., 2018).

OrganoSeg2 was released in 2026 (Wells C.J. et al., 2026). The new version achieves a tenfold increase in data processing speed compared to previous version, introduces new organoid segmentation settings with improvements in accuracy and robustness, and enables dynamic studies. However, like OrganoSeg, OrganoSeg2 is not fully autonomous: manual corrections remain necessary for overlapping spheroids. Parameter configuration can be challenging for beginners, and detection performance for small, dense organoids is inferior to that for larger ones.

### 6.2 Morphometric analysis of intestinal organoids *in silico* и *in vitro*

This approach combines mathematical models with experimental data to assess the development and morphological changes of intestinal organoids, such as crypt formation and depth, as opposed to subjective visual assessment. It combines crypt-counting algorithms with agent-based models to simulate biomechanical interactions (e.g., stem cell stiffness and its impact on morphogenesis) and predict intestinal crypt structure, deepening our understanding of tissue formation and homeostasis. This approach provides tools for quantifying crypt-like structures at days 3–7 of culture, helping to assess maturity and study drug response. A key advantage is its support for *in silico* studies, which enable the testing of hypotheses about cell behavior and morphogenesis, reducing the need for expensive and labor-intensive *in vitro* experiments. One of the limitations is that the accuracy of morphometric analysis relies heavily on the quality of image segmentation, requiring either manual segmentation (which is time-consuming) or automated algorithms, which may require refinement. Current approaches often focus on 2D projections or cross-sections of organoids, limiting their applicability for the full and detailed study of complex 3D structures. Morphometric analysis of intestinal organoids *in silico* and *in vitro* represents an important step toward modeling and quantifying morphogenesis, demonstrating how the integration of biology and computation can deepen our understanding of tissue development. The ability to predict organoid structure and behavior based on mathematical models not only reduces experimental costs but also allows us to explore mechanisms that are difficult to study solely *in vitro*. However, the dependence on segmentation quality and the limitations of 3D modeling indicate the need for further development of algorithms and computational power to fully realize the potential of this approach (Montes-Olivas et al., 2023). Furthermore, morphometric models based on deep learning algorithms are currently being developed and used within integrated tools (pipelines), such as Deliod. This tool has a modular architecture and allows not only for morphometric analysis *per se* but also for determining the organoid class to which the object in question belongs, as well as the organoid’s stage of development, i.e., the complexity of its organization (Sun et al., 2025).

### 6.3 AMIDA (automated morphometric image data analysis)

AMIDA enables the automatic analysis of large datasets, including thousands of multicellular structures. The program processes various spheroid shapes (round, star-shaped, invasive) and measures size, symmetry, and texture parameters such as area, circularity, roughness, density, and others (19 parameters in total). In addition to basic viability, AMIDA evaluates dynamic morphological changes: invasion patterns, epithelial-mesenchymal transition, and cytoskeletal reorganization.

AMIDA results are confirmed by 2D proliferation/apoptosis assays and manual analysis. The program is optimized for 3D cultures in matrices (e.g., Matrigel), which capture physiologically significant features such as lumen formation and basement membrane integrity.

AMIDA’s limitations include the skill required to configure segmentation algorithms and analysis parameters, especially for structures with a wide range of sizes or to minimize matrix artifacts. Accuracy depends on the quality of microscopy and standardized protocols. AMIDA focuses on multicellular structures, which can mask the heterogeneity of subpopulations within spheroids, limiting the study of individual cell dynamics. The program places high demands on computational power for analyzing large volumes of images. The multiparameter AMIDA program is a powerful tool for comprehensive phenotypic screening of 3D cultures, enabling the quantification of not only basic morphological parameters but also dynamic changes associated with biological processes. Its ability to handle thousands of samples and matrices makes it valuable for high-throughput studies. However, as with other complex tools, the need for calibration and customization for specific tasks, as well as the high computational demands, highlight the importance of specialized knowledge and appropriate infrastructure for effective use (Härmä et al., 2014; Piccinini, 2015).

### 6.4 MOrgAna

MOrgAna provides a user-friendly interface for non-experts and modular Python code for advanced users, enabling rapid processing of hundreds of images. The program outperforms algorithms such as OrganoSeg and CellProfiler in pixel classification (Jacquard coefficient metrics, accuracy), using deep learning for complex contours. MOrgAna is able to distinguish between background, organoids, and their boundaries, improving detection in environments with high cellular debris content.

However, MOrgAna requires manual image annotation for training. It is best suited for environments with a single organoid per image, which complicates the analysis of dense cultures. The program also requires high computing power from GPUs to process large volumes of 3D images. MOrgAna demonstrates the potential of deep learning for highly accurate segmentation of complex organoid contours, especially in conditions where traditional algorithms may be less effective. Its advantage in classifying pixels and recognizing fine boundaries suggests its potential to overcome challenges associated with cellular deficiencies and environmental heterogeneity. However, the need for manual labeling for training and the limitations of analyzing dense cultures highlight the tradeoff between the high accuracy achieved through deep learning and the labor-intensive nature of data preparation. This highlights the ongoing need to develop methods that require less manual intervention (Gritti et al., 2021; Bai et al., 2024).

### 6.5 Additional tools and platforms

In addition to the above, a number of other tools and platforms are making significant contributions to organoid morphometry and bioimage analysis in general.

BrAIn (Brain Organoid AI Platform) is designed for high-throughput morphological analysis of brain organoids. It uses deep learning to quantify complex structural features and dynamic changes, enabling standardized automated characterization of organoid development. The program enables the reading and analysis of morphometric parameters such as area, Feret diameter, perimeter, and circularity. Validation demonstrates its accuracy in identifying subtle phenotypes and predicting functional outcomes (Kahveci et al., 2025).

OrganoLabeler is a deep learning-based tool designed for rapid and accurate labeling of organoid images. This simplifies the analysis of morphology, growth, and differentiation using an intuitive interface and automatic segmentation, reducing the labor required for manual labeling while maintaining high accuracy (Kahveci et al., 2024).

OrganoID is an AI-based platform that enables the automatic tracking and quantification of individual organoids over time. It combines computer vision and deep learning to monitor morphology, growth, and movement. OrganoID enables the estimation of organoid area, perimeter, circularity, organoid-to-convex hull ratio, and elliptical deviation from circularity (Matthews et al., 2022; Alieva et al., 2024).

LabelFreeTracker is a fluorescence-free imaging method for tracking cells in organoids using 3D bright-field imaging. The use of fluorescent labels in living systems can be phototoxic, alter cell behavior, and limit the duration of experiments. Therefore, the authors of the article are exploring the possibility of obtaining fluorescence-free images. LabelFreeTracker uses a convolutional neural network (3D U-net) to predict three-dimensional cell and nuclear boundaries based on bright-field images. Using this method, the following parameters can be extracted: cell number, cell volume (Kok et al., 2025).

The development of artificial intelligence platforms such as BrAIn, OrganoLabeler, and OrganoID demonstrates the shift from specialized tasks to comprehensive solutions in organoid morphometry. These deep learning-based tools automate routine and labor-intensive processes such as labeling and tracking, significantly increasing research productivity. Their ability to standardize analysis and identify subtle phenotypes highlights the growing role of artificial intelligence in achieving the high accuracy and scalability required for modern biological research.

Ilastik is an interactive open-source software developed for analyzing biological images using machine learning. It enables users without advanced computer skills to solve segmentation, object classification, counting, and tracking tasks in images. Ilastik allows users to train models on their own data directly within the program, without the need to write code, which is advantageous for specific biological objects, but requires image labeling and time. It supports random forest and other classifiers from the scikit-learn library and handles images up to 5D (3D + time + channels) (Berg et al., 2019; Stringer et al., 2021).

CellProfiler is open-source software developed for the automated analysis of biological images to identify and quantify cellular phenotypes. This enables the processing of hundreds of thousands of images, which is crucial for large-scale screening. CellProfiler uses the concept of “pipelines” (sequences of modules) to customize specific tasks and includes over 50 modules for various analysis steps, including illumination correction, object segmentation, and parameter measurement (size, shape, intensity) (Stringer et al., 2021; McQuin et al., 2018; Åkerfelt et al., 2017).

Fiji is a distribution of the popular open-source software ImageJ, specifically designed for biological image analysis. The core idea of Fiji is to integrate powerful software libraries with a wide range of scripting languages, enabling the rapid prototyping of image processing algorithms. Fiji aims to simplify the translation of new algorithms into ImageJ plugins, which can then be easily distributed to end users through an integrated update system. The authors position Fiji as a platform for productive collaboration between the computer science and biology research communities, highlighting the growing need among biologists for computational solutions to handle the ever-increasing volume and complexity of images obtained using automated microscopy technologies (Schindelin et al., 2012; von Chamier et al., 2021; Schraivogel et al., 2022).

AnaSP is an open-source software for automatic estimation of morphological parameters of spheroids based on light-field images obtained with a standard wide-field microscope. This software can be used to extract parameters such as minor and major axes, equivalent diameter, perimeter, area, volume, and sphericity (Piccinini, 2015; Piccinini et al., 2017; Zanoni et al., 2016). Tools such as Ilastik and CellProfiler demonstrate the importance of extending the capabilities of existing platforms and general-purpose machine learning tools. Ilastik and CellProfiler, in turn, provide flexible solutions for bioimage analysis, allowing researchers without advanced programming skills to apply machine learning methods. However, the versatility of these tools may mean that specific organoid morphometry tasks require additional model tuning and training, which is a trade-off between flexibility and specificity.

OrganoidTracker is a semi-automated tool for tracking cells in organoids using machine learning and manual error correction. Cell detection is performed using a neural network (3D U-Net architecture) trained on manual annotations of cell nuclei centers. A low-overhead flow detection algorithm is used to construct cell trajectories, optimizing for motion, nuclear volume changes, and track length. The program automatically flags suspicious events, but the user only needs to check and correct ∼1–2% of the data, significantly speeding up the process compared to fully manual tracking (Kok et al., 2020).

TrackMate is an open-source and extensible plugin for automatic, semi-automated, and manual tracking of individual particles in time-series images, developed as a Fiji/ImageJ plugin. It can handle 2D, 3D, and 4D images and can be customized by adding dedicated tracking, detection, visualization, or analysis modules (Tinevez et al., 2017; Moen et al., 2019; Rueden et al., 2017). These methods emphasize the shift from static morphometry to dynamic analysis and tracking of cellular kinetics within organoids. The ability to track the movement, division, and disappearance of individual cells in 3D time-series images enables the study of tissue growth and homeostasis at the single-cell level. A semi-automated approach combining machine learning with human correction achieves high accuracy with significantly reduced labor costs, which is critical for understanding dynamic processes in developing organoids responding to stimuli. In addition to the methods and algorithms discussed above, the following studies, which are not yet actively used, can be applied to cell image processing:

SpheroidAnalyseR is a web-based platform designed for automated analysis of data obtained from experiments with 3D spheroids or organoids grown in 96-well plates. SpheroidAnalyseR provides an intuitive online tool that allows users to upload raw images, analyze them, and obtain quantitative results for each spheroid/organoid. Designed for scientists without programming experience, the platform automates routine size measurement processes, making it a valuable tool for drug screening and other high-throughput studies. SpheroidAnalyseR can analyze parameters such as object area, diameter, and circularity (Barrow et al., 2022).

### 6.6 The comparative advantages and limitations of the discussed morphological tools

This chapter presents the advantages and limitations of morphometric tools in the form of two tables. Table 1 presents a comparative assessment (Score) of morphometric instruments and Table 2 deciphers the criteria for this assessment. To facilitate the comparative assessment, a summary table was compiled. It should be noted that rigorous ranking of software packages requires testing them on a single, uniform dataset–including identical cell lines, organoid types, and image acquisition parameters. In the present review, such validation was not performed; all data presented are obtained exclusively from published sources.

**TABLE 2:** Criteria for evaluating the performance of the automated program.

Criteria	0	1	2
А (Automatization)	Manual using	Semi-automated	Automated
С (User complexity)	High	Average	Low
F (Number of Analyzed Features)	Only segmentation	1–10 Analyzed Features	More than 10 Analyzed Features
M (Microscopy method availability)	Laser microscopy	Confocal, fluorescence microscopy	Bright field microscopy

After analyzing the measured parameters of the presented instruments (see Table 1), the following common parameters were identified for monitoring organoid growth: Area, Perimeter, Diameter. All of these parameters are measurable qualitatively by daily visual monitoring of organoid growth over a long period of time. Therefore, the importance of converting these parameters from a qualitative scale to a quantitative assessment increases (Serafini et al., 2024). For example, Serafini et al. measured and compared organoid diameter as an indicator of their growth. The research team also measured the roundness, density, and aspect ratio of the organoids to characterize their shape. Differences were observed early in organogenesis, although no statistically significant differences were observed in these parameters between healthy people cerebral organoids and cerebral organoids with a mutation in the gene TSC. Castiglione et al. proposed a standardized method for quality control of cortical organoids (cultured for 60 days). This non-invasive, five-point scoring method allows for the screening of low-quality organoids (Castiglione et al., 2025).

It should be noted to develop criteria for a comprehensive assessment of organoids at the cellular, histological, functional, and morphological levels, it is first necessary to identify organoid quality standards at each of these levels. Morphometric methods allow for the identification of such standards at the morphological level. Furthermore, these methods are noninvasive and easy to use compared to functional tests (electrical activity recording using MEA, dynamic assessment using Сalcium imaging, optogenetics, biocomputational tests, and synaptic plasticity analysis) and single-cell RNA sequencing (scRNA-seq). This allows for the use of morphometric methods over long time to monitor organoid growth and development, as well as to observe changes in morphometric parameters to track change patterns.

### 6.7 Additional tools for cell structures analysis

The following articles analyzed brain cell structures and their morphometric parameters.

Yakovlev E. V. et al. presented a machine-learning-based approach for the recognition and morphological analysis of unlabeled astrocytes imaged using phase-contrast microscopy. Astrocytes play a key role in brain function and have complex, branched morphology. Their analysis is often performed using fluorescence microscopy, which can impact cell viability and data accuracy due to fixation and staining (Yakovlev et al., 2024; Lenk et al., 2020; Zhuo et al., 1997). Phase-contrast microscopy allows for the study of cells without exposure, but produces low-contrast images, complicating subsequent processing. The proposed machine-learning approach aims to overcome these limitations, enabling the temporal evolution of astrocyte morphology to be tracked and quantitative data on their properties to be obtained. This approach allows for the extraction of the following quantitative parameters from astrocyte images: cell area, nuclear size, astrocyte branch length, and node count (Yakovlev et al., 2024).

BCSnet is a deep learning model based on the U-Net architecture, developed for automatic segmentation of brain cells in trypan blue-stained images. BCSnet provides an automated and robust segmentation method that can be used to analyze cell viability. The study found that the best model had a Soren-Dice index of 0.9189. The trained neural network allows for the assessment of live neurons three orders of magnitude faster than manual calculations and with an accuracy comparable to histological accuracy. According to the study, the difference between live and dead cells can be identified by parameters such as chromatin morphology, cell area, and nuclear shape (Kudryavtsev et al., 2024).

## 7 Modern trends and prospects for the development spheroids and organoids morphometry methods

Current trends in organoid morphometry are characterized by a drive for increased automation, resolution, speed, and data integration to achieve a more comprehensive and dynamic understanding of biological processes. The development of visualization methods and software is moving toward integrated platforms capable of processing large volumes of data with minimal human intervention.

One key area is the ongoing development and implementation of artificial intelligence (AI) and machine learning. This includes the creation of more sophisticated neural networks for organoid segmentation and classification, capable of handling high heterogeneity and complex morphology, as well as for the automatic identification of subtle phenotypes and prediction of biological responses. Platforms such as BrAIn, OrganoLabeler, and OrganoID are already demonstrating the potential of AI to standardize and accelerate analysis, as well as to track dynamic changes. Future developments will focus on creating models that require less manual annotation and are capable of self-training on large, unlabeled datasets.

A second important trend is the integration of multivariate data. This means integrating morphometric data obtained using various microscopy techniques (for example, light sheet microscopy for dynamics, and transmission electron microscopy for ultrastructure) with other types of biological data, such as transcriptomics, proteomics, or metabolomics. This synergy of methods will enable comprehensive analyses linking morphological changes to their underlying molecular mechanisms. This will lead to a deeper understanding of the functional morphology of organoids and their response to various stimuli.

The third area is high-throughput 4D analysis. The need to dynamically study organoids, tracking their growth, differentiation, cell migration, and response to stimuli in real time requires further development of methods that combine high-speed imaging with minimal phototoxicity. Light microscopy is already a leader in this field, and future innovations will focus on improving its resolution, penetration depth, and ability to work with larger and more optically dense samples. The development of tools for automated cell tracking in organoids, such as OrganoidTracker and TrackMate, will be crucial for the quantitative assessment of cellular kinetics.

The fourth trend is standardization and accessibility. The development and dissemination of open-source software solutions (e.g., ilastik, CellProfiler) and standardized morphometric analysis protocols will improve the reproducibility of research results and facilitate data exchange between laboratories. This also includes the creation of intuitive user interfaces that will allow researchers without advanced programming skills to effectively use complex computational tools.

Finally, future prospects include the creation of “digital twins” for organoids, which will combine experimental data with mathematical models to predict behavior and response to treatment. This has already been demonstrated in the analysis of intestinal organoids *in silico* and *in vitro*. Such approaches will facilitate more efficient experimental design and reduce costs. The future of organoid morphometry lies in the synergy of methods, where various imaging technologies and computational tools will be integrated to create comprehensive analytical platforms. This will overcome existing technological barriers and open new horizons for biological research, from fundamental understanding of organ development to the development of personalized therapeutic strategies. Overcoming the limitations of individual methods through their combination and the use of advanced computational approaches will enable the extraction of unprecedented amounts of information from organoid models, ultimately accelerating the translation of scientific discoveries into clinical practice.

## 8 Conclusion

A review of the literature on organoid morphometry demonstrates the rapid development of this field, driven by the growing importance of 3D and 4D cell models in biomedical research. The transition from simple two-dimensional cultures to complex three-dimensional structures has required the development of fundamentally new approaches to visualization and quantitative analysis.

In parallel with the development of visualization methods, software tools for segmentation and morphometric assessment are rapidly advancing. Tools such as OrganoSeg, AMIDA, and MOrgAna demonstrate increasing automation and scalability of analysis, allowing the processing of thousands of organoids and the extraction of numerous parameters. The use of deep learning in platforms such as BrAIn, OrganoLabeler, and others significantly improves segmentation accuracy and automates routine processes, although it often requires preliminary manual data annotation. The development of universal tools (ilastik, CellProfiler) and specialized plugins (TrackMate) expands the accessibility and flexibility of analysis, allowing researchers to tailor solutions to their needs. Overall, the field of organoid morphometry is on the cusp of significant breakthroughs that will enable more accurate and efficient use of these unique biological models for fundamental discoveries and translational applications in medicine.
